# Current development of biosensing technologies towards diagnosis of mental diseases

**DOI:** 10.3389/fbioe.2023.1190211

**Published:** 2023-06-29

**Authors:** Yuhan Zheng, Chen Liu, Nai Yeen Gavin Lai, Qingfeng Wang, Qinghua Xia, Xu Sun, Sheng Zhang

**Affiliations:** ^1^ Faculty of Science and Engineering, University of Nottingham Ningbo China, Ningbo, China; ^2^ Ningbo Research Center, Ningbo Innovation Center, Zhejiang University, Ningbo, China; ^3^ Robotics Institute, Ningbo University of Technology, Ningbo, China; ^4^ Nottingham Ningbo China Beacons of Excellence Research and Innovation Institute, University of Nottingham Ningbo China, Ningbo, China; ^5^ School of Mechanical Engineering, Zhejiang University, Hangzhou, China

**Keywords:** biosensors, mental diseases, eye-tracking, EEG signals, EOG signals, virtual reality, diagnosis method

## Abstract

The biosensor is an instrument that converts the concentration of biomarkers into electrical signals for detection. Biosensing technology is non-invasive, lightweight, automated, and biocompatible in nature. These features have significantly advanced medical diagnosis, particularly in the diagnosis of mental disorder in recent years. The traditional method of diagnosing mental disorders is time-intensive, expensive, and subject to individual interpretation. It involves a combination of the clinical experience by the psychiatrist and the physical symptoms and self-reported scales provided by the patient. Biosensors on the other hand can objectively and continually detect disease states by monitoring abnormal data in biomarkers. Hence, this paper reviews the application of biosensors in the detection of mental diseases, and the diagnostic methods are divided into five sub-themes of biosensors based on vision, EEG signal, EOG signal, and multi-signal. A prospective application in clinical diagnosis is also discussed.

## 1 Introduction

Biosensors are instruments that apply bio-sensing elements to collect information recorded through specific biological, physical, and chemical changes, which are converted into measurable signals (Vigneshvar et al., 2016). These properties that can be detected include changes in pH, gas, mass, electron transport, heat transport, and absorption and release of specific ions (Velasco-Garcia and Mottram, 2003; Zhang et al., 2021a). Biosensors have been utilized successfully in many areas, such as biological signal monitoring, environmental surveying, motion observation, gas analysis, health tracking (Zhang et al., 2021a; Liu et al., 2021; Zhang et al., 2022), and in the field of medical applications and healthcare (Guo et al., 2021; Wang et al., 2022). The advancement of biotechnology and new materials have paved the way for the development of advance biosensors that aid the detection of mental diseases (Zhang et al., 2021b). Being noninvasive, low-cost, wearable, sensitive, and dynamic in monitoring, endow the detection technologies with increasing accuracy, response rate, deformability, and biocompatibility (Guo et al., 2019; Yang et al., 2019). This makes them of great value for healthcare practitioners in the treatment of mental diseases.

Mental diseases are disorders that affect cognition, emotion, volition, and behaviors (Hyman et al., 2006). Episodes of mental disease can severely interfere with learning and social skills. Mental diseases often begin early in life and are often chronic relapsing processes (Hyman et al., 2006). According to the World Mental Health Report, about 1 billion people worldwide have mental diseases (Freeman, 2022). The outbreak of COVID-19 has also exacerbated this phenomenon (Li et al., 2020). It is difficult for some patients with mental diseases and chronic diseases to receive continuous treatment during COVID-19, which may lead to relapse of mental diseases and exacerbation of negative emotions (Psychiatry CSo, 2020; Bo et al., 2021). Additionally, symptoms of depression and anxiety have also increased in children and adolescents (Racine et al., 2020). Therefore, research related to mental diseases has received increasing attention. There is a wide range of mental diseases, including Alzheimer’s disease, depression, schizophrenia, autism spectrum disorder, and various personality disorders (Busfield, 2011). The traditional diagnosis of mental diseases requires multiple physical indicators, combined with self-rating scales and nursing reports from guardians and psychiatrists (Möricke et al., 2016; Römhild et al., 2018). Furthermore, the bias of subjective judgment may lead to misdiagnosis, which affects the treatment of the diseases (Huang et al., 2017). Therefore, diagnosing mental disorders around the world is difficult. Compared with traditional diagnostic techniques, biosensors can quantify the qualitative expression of the brain by detecting biomarkers, and avoid cultural and language differences of the subjects (Hidalgo-Mazzei et al., 2018).

Biomarkers are defined as biological characteristics measurable in biological media (such as human tissues, cells, or fluids) as indicators of normal biological processes, pathogenic processes, or pharmacological responses to therapeutic interventions (Mayeux, 2004). Mental diseases are often accompanied by the impairment of some functions, e.g., impaired visual attention processing, social dysfunction, and restrictive, repetitive behaviors (Schmidt et al., 2011; Edition FJAPA, 2013). Abnormality of these biomarkers can be measured by biosensors to distinguish the presence and absence of disease states (Schmidt et al., 2011), which provides a more convenient and effective tool for rapid diagnosis. These bio-sensors utilize cutting-edge technology to not only collect and compare biological data, such as eye-tracking data, electroencephalography (EEG), electrooculogram (EOG), and cognition and behavior in virtual reality (VR), but also employ machine learning algorithms to extract biometrics for the more objective results and higher accuracy (Burdea and Coiffet, 2003; Plitt et al., 2015; Ibrahim et al., 2018; Yaneva et al., 2018; Yamagata et al., 2019; Zhao et al., 2021). The multiple-signal sensors combined with machine learning were found to be a relatively novel trend, which offer a more comprehensive understanding of participant responses. However, the pre-preparation and collection operations of a single biosensor are already relatively cumbersome. The popularization of large-scale mental disease screening and telemedicine requires a more efficient, higher-precision and wearable multi-signal integrated sensor in the future.

This review summarizes the research trend of biosensors towards the detection of mental diseases. The review focuses on five objective quantification methods of the field, namely vision-based, EEG signal, EOG signal, VR-based, and multiple-signal sensors. Vision-based sensors detect mental diseases associated with abnormal visual attention through eye-tracking devices. EEG and EOG signals can detect mental diseases associated with abnormal brain and eye signals. In addition, VR-based sensors offer more possibilities for detecting mental disease associated with impaired spatial navigation and memory. The use of multiple-signal sensor can improve diagnosis accuracy and efficiency. The review also describes detection devices, signal processing methods, and disease assessment techniques in detail. The future research directions and application prospects are also covered in this review.

## 2 Mental diseases

### 2.1 AD (Alzheimer’s disease) and MCI (mild cognitive impairment)

Alzheimer’s disease, whose chief symptoms are memory impairment, attention deficit, and executive dysfunction, is one of the most common neurodegenerative diseases worldwide that lead to dementia (Tschanz et al., 2006; Belleville et al., 2007). According to the Alzheimer’s Association, more than 10 percent of people over 65 in the United States suffer from this kind of disease, and the proportion reaches nearly half among people over 85 (Meek et al., 1998). As the ageing of the population, the prevalence of Alzheimer’s disease is predicted to increase twice before 2050 (Mattson, 2004). However, until now, there is still no effective cure for Alzheimer’s disease, and medicines can relieve the symptoms to some extent (Dauwels et al., 2010). Mild cognitive impairment (MCI) is the intermediate stage between the cognitive decline associated with normal ageing and early AD (Gauthier et al., 2006). Hence, early detection of MCI is significant in preventing AD. The traditional diagnostic methods for Alzheimer’s disease need to combine other techniques, including medicinal history analysis, neurological tests, blood tests, and psychological tests, which are highly tedious, subjective, and expensive (Sunderland et al., 2006; Weiner MJTjon et al., 2009). Currently, more efficient and accurate diagnostic approaches are badly needed.

### 2.2 ASD (autism spectrum disorder)

Autism spectrum disorder is a neurodevelopmental disorder characterized by social communication disorders and repetitive behaviors (Edition FJAPA, 2013). Autism affects more than 70 million people worldwide, and about 1 in 68 children suffer from this disorder (Wang et al., 2013). The cause of ASD is still unknown. According to available scientific evidence from the World Health Organization, many factors can lead to ASD in children, including abnormal brain development and neural reorganization in early childhood (Black et al., 2017). O date, the diagnosis of ASD has typically been based on observation of daily functionality and behavioral characteristics of patients. The Diagnostic and Statistical Manual for Autism (DSM-V) provides diagnostic criteria for referencing clinical symptoms. Other ancillary assessment tools, including behavioral scale tests, checklists, and questionnaires, supplement diagnostic results (PEREIRA et al., 2007). The standards to diagnose ASD are incredibly complex and diverse, resulting in dysfunctions with variation among individuals. Hence, these evaluation methods are not objective due to no reliable biomarkers. Furthermore, more accurate and objective clinical detection methods towards ASD are badly needed.

### 2.3 Depression

Depression is a common mood disorder. The data published by the World Health Organization showed that more than 300 million people worldwide suffer from depression (Organization, 2017). Depression results from various factors, including the interaction of social, psychological, and biological factors, and thus the causes of depression are complex. Depression is different from short-lived sadness in everyday life. The process of depression is recurrent, including periods with symptomatic episodes and periods of recovery. During a depressive episode, patients feel a low mood, anhedonia, and low energy. In the worst cases, depression can even lead to suicide. The people who experienced depressed moods almost every day or for at least 2 weeks were diagnosed with depression according to the DSM-IV classification (Wong and Licinio, 2001). However, in developing countries, people with depression are often not detected as early as possible (Chisholm et al., 2016). To this end, the researchers are trying to identify biometric markers that could be used to distinguish depression patients from healthy individuals in primary care. Among the biometric markers, attention bias has become the optimal marker and has been applied in various works. Studies of attention bias in clinical disorders have inferred attention bias by assessing reaction time. Studies have shown that people with depression focus more on negative stimuli than positive ones (Klawohn et al., 2020).

### 2.4 Schizophrenia

Schizophrenia is a multi-attribute chronic disease, resulting in sensory perception, thinking, emotion, volitional behavior, and cognitive dysfunction in the brain. These symptoms vary widely among patients. Existing instruments are not powerful enough to fully assess the mental states of patients. The traditional detection of schizophrenia is based on a diagnosis from the psychiatrist and supplemented by Computed Tomography (CT) diagnosis, Magnetic Resonance Imageing (MRI) diagnosis, and Positron Emission Computed Tomography (PET) diagnosis (Andreasen, 1995). Therefore, detection methods independent of schizophrenic drugs and the mental states of patients are being explored.

### 2.5 Sleep disorder

Sleep quality is an essential indicator for assessing human homeostasis. Sleep disorder is a kind of mental disease associated with severe medical, psychological, and social obstacles (Silber et al., 2007). Another widely used method is polysomnographic (PSG), which uses a variety of electrodes and sensors to record about ten signals during sleep (Yildirim et al., 2019). This method is complex and expensive. Therefore, it is necessary to develop a new automated sleep disorder detection system that can help doctors assess the sleep stages of patients more efficiently and accurately. When people fall asleep, their eye movements tend to slow down and their EOG values tend to be lower (Carskadon and Dement, 2005). The EOG signals help distinguish between rapid eye movement and non-rapid eye movement. Due to the significant progress made in object detection by deep learning technology (LeCun et al., 2015), various subjective diagnosis methods based on the EOG signals have been proposed.

### 2.6 Epilepsy

Epilepsy is a chronic brain disease in which neuronal activity is abnormally synchronized or excessive, characterized by transient and recurrent seizures. Frontal lobe epilepsy (TLE) is a standard focal epilepsy diagnosed by advanced imaging examination and EEG. Most TLE patients are associated with memory deficit and attention disorder (Blackwood et al., 1994; Bocquillon et al., 2009). Traditional diagnosis of epilepsy is based on patient and eyewitness descriptions and video recordings of seizures. Unfortunately, it is common for seizures to be misdiagnosed as other disorders, e.g., convulsive syncope (Bocquillon et al., 2009). Consequently, there is a need for an accurate way to discriminate epilepsy.

Vision-based sensors can detect AD, ASD and depression. Depression and ASD also can be diagnosed by EEG sensors, as well as epilepsy, schizophrenia, and epilepsy. EOG sensors are capable of diagnosing schizophrenia and sleep disorders which are detectable by EEG signal sensors. VR-based sensors can detect mental diseases including all of the above except sleep disorders. Multiple-signal sensors can diagnose all of the above mental diseases.

## 3 Applications

### 3.1 Vision-based sensors

**FIGURE 1:**
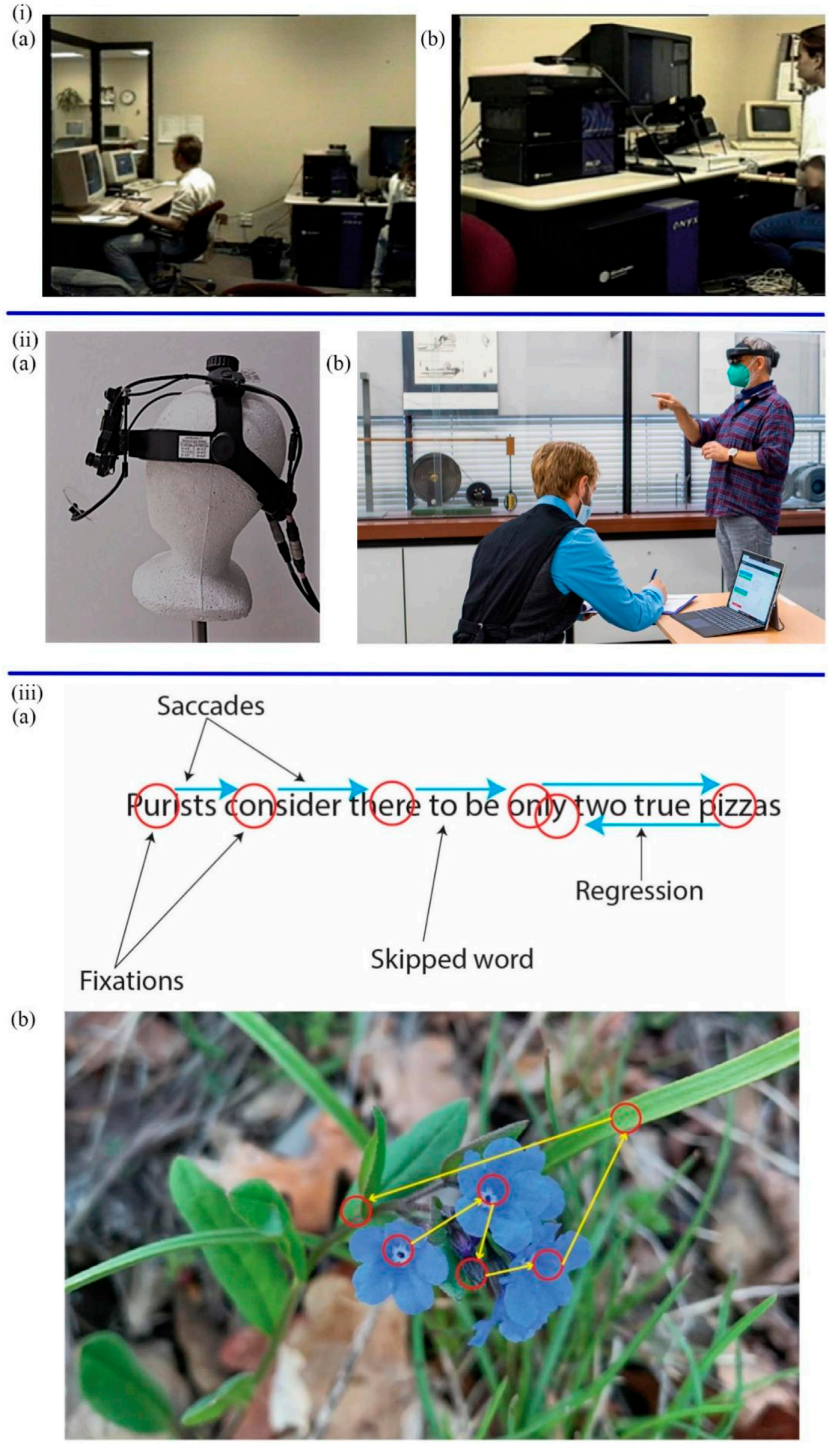
**(i)** The portable video-based eye-tracker **(A)** The operator **(B)** The Participant (Hutton, 2019). **(iiA)** The head-mounted video-based eye-tracker (Gotardi et al., 2020). **(B)** The participant wears the eye-tracker (Kapp et al., 2021). **(iiiA)** The forms of eye movements in the reading scene **(B)** The saccades and fixations in images (Carter and Luke, 2020).

Eye-tracking is the process of measuring individual eye movements and gaze positions to reflect gaze behavior. Usually, when a person gazes at an object, attention is shifted to a specific point in order to be able to examine in detail the image occupying the direction of the gaze center (Kennedy, 2016). As shown in Figure 1, the prevalently used eye-tracking technologies are table-mounted and head-mounted video-based eye-trackers (Hutton, 2019; Carter and Luke, 2020). The eye trackers can record forms of eye movements, including fixations, saccades, and other types (blinks, smooth pursuits, and vergence) by measuring the position of infrared corneal reflection relative to the pupil (Rayner, 2009; Carter and Luke, 2020). In the eye-tracking task, participants are required to gaze at images (i.e., pictures, videos, and web pages) (Zhao et al., 2021) in order to provide information about the attention allocation of a person in a visual scene (Ashraf et al., 2018). Multiple attempts to diagnose mental diseases based on eye-tracking technologies have shown a bright future in clinical diagnosis.

**FIGURE 2:**
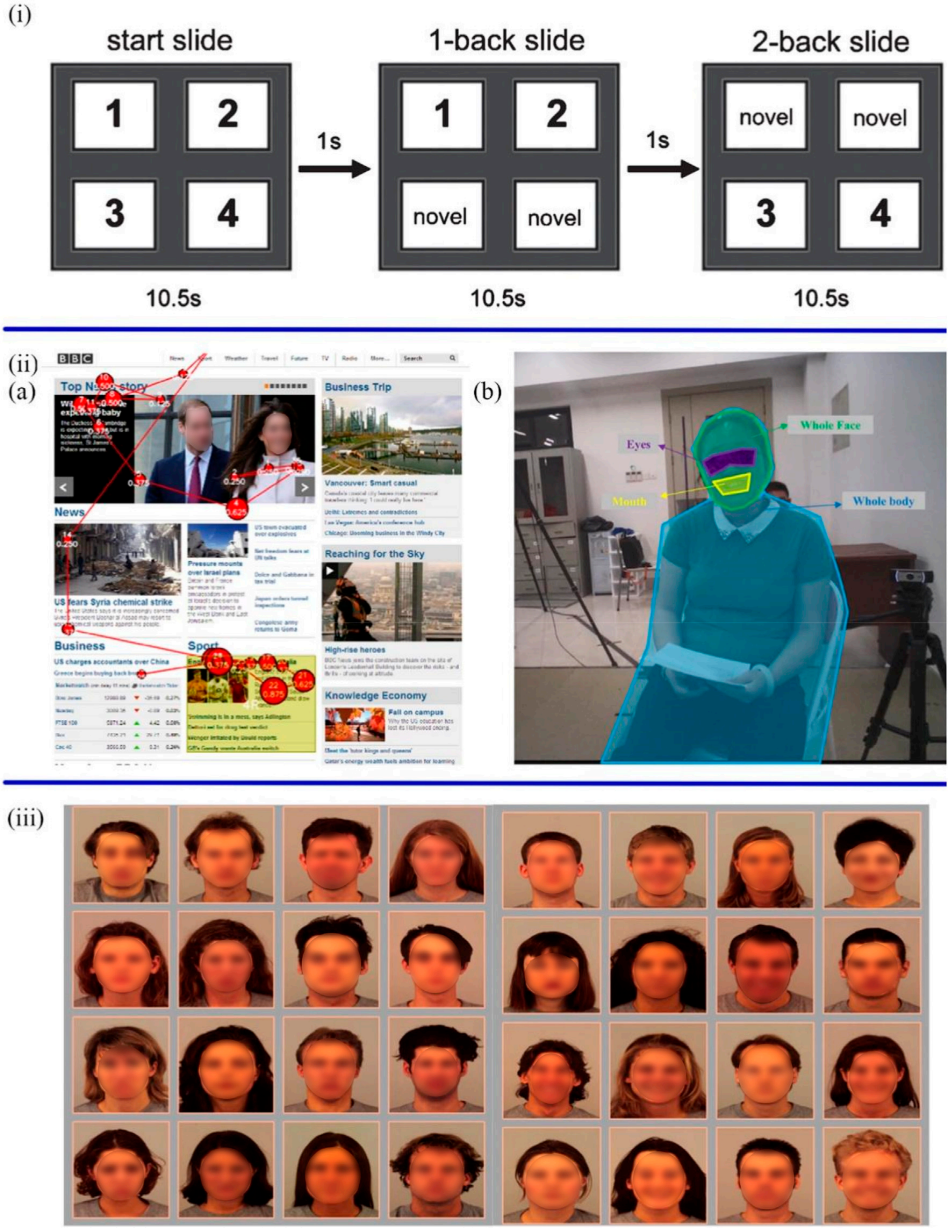
**(i)** The slides started with four new images, and after 10.5 s switched to the next slide with two new images and two duplicate images (Chau et al., 2017). **(iiA)** The scan path of ASD participants during the task of reading the BBC website (Yaneva et al., 2018). **(B)** The analysis of visual gaze features is based on four areas of interest, including eyes, mouth, whole face and whole body (Zhao et al., 2021). **(iii)** The sad and neutral facial expressions in the left 4 × 4 grid and the happy and neutral facial expressions in right 4 × 4 grid (Klawohn et al., 2020).

The selective attention is defined as the ability to screen out relevant and applicable information (Levinoff et al., 2004). Given that selective attention, associated with activity in neurotransmitter function, is impaired in the early stages of AD (Perry and Hodges, 1999; Rangel-Gomez and MJJop, 2016). The study of selective attention to new stimuli can provide new insight into the cognition and attention of AD patients. Chau et al. proposed a non-verbal and non-invasive diagnostic method to predict cognitive decline from mild to moderate AD patients by estimating the novelty preference of patients (Chau et al., 2017). The binocular eye-tracking systems recorded the eye gaze data, including the position, time, and frequency when AD patients viewed novel and repeated images on slides (Figures 2i. These visual scanning parameters were processed by automatic classification algorithms to estimate novelty preference. Standardized Mini-Mental Status Examination (SMMSE) and Conners Continuous Performance Test (CPT) were used to assess the cognition and attention of patients separately. The results showed that impaired people have less attention or preference for novel images than the control group.

Yaneva et al. (2018) described an unobtrusive method for detecting ASD through two everyday tasks: browsing and searching web information (Figures 2ii,A). Six web pages were randomly presented to 36 participants (16 ASD patients and 16 non-ASD controls) and their gaze data were collected by an eye-tracker in 2 minutes. Five gaze features (i.e., time viewed %, time viewed sec, time to first view, fixations and revisits) and five non-gaze features (i.e., participant gender, AOI ID, correct answer AOI, media ID, and level of visual complexity) were used to train a machine learning classifier to identify whether participants had ASD by Logistic Regression (LR) with 100-fold cross-validation. The basic principle of this method is to reveal the different attention-shifting mechanisms of two groups based on the new alternative marker of attention differences. The preliminary result showed that the search task elicited higher differences between the two groups than the browsing task. The classifier achieved 0.75 classification accuracy, which could be proved effective in detecting ASD. In addition to the web task, Zhao et al. (2021) proposed another approach for diagnosing ASD based on eye-tracking data from face-to-face conversations. Participants wearing a head-mounted eye-tracker had a conversation with a female interviewer (see Figures 2iiB). The four arranged informal sessions included general questions, hobbies, yes-no questions, and questions from respondents. Afterwards, the visual fixation features and session length features from the eye-tracking data were extracted to determine which of the four machine learning classifiers had optimal classification accuracy by implementing forward feature selection. The classifiers include Support Vector Machine (SVM), Linear Discriminant Analysis, Decision Tree, and Random Forest (RF). The results proved that all classifiers reached a maximum classification accuracy of more than 84%, and the SVM classifier achieved the highest classification accuracy of 92.31%. Consequently, children with ASD can be preliminarily diagnosed in daily life through face-to-face conversations.

Sanchez et al. further elaborated on the role of attentional bias in diagnosing depression (Sanchez et al., 2013). They designed an eye-tracking task to evaluate the disengagement of attention from emotional stimuli and test whether mood changes during and after stimulation in depressed patients are associated with visual dissociation difficulties. 19 participants with Major Depression Disorder and 16 healthy participants were free to view emotional (i.e., happy, sad, and angry) and neutral faces. Eye-tracking devices synchronously record the initial orientation, fixation frequency, and fixation time during 3,000 ms. In the subsequent engagement-disengagement task, an attention interval was also recorded when moving from emotional to neutral faces. Studies have found that people with depression took longer to eliminate depression-related emotions, e.g., sad faces. In other words, with the increase in depression, the disengagement from negative information becomes slow. Therefore, negative attention disengagement difficulties can be regarded as a critical feature of depression. Ferrari et al. replicated the above Sanchez experiment and further proposed the attentional bias modification (ABM) tasks (Ferrari et al., 2016). The ABM tasks, containing positive training (PT) and negative training (NT), were designed to evaluate and train the components that interfere with attention in depression. The participants included 78 female and 17 male college students and were assigned to PT (n = 48) or NT (n = 47) in a double-blind fashion. In the PT, the test continued when the participants separated from negative pictures and gazed at the positive pictures for 1000 ms. In contrast, in the NT, participants were asked to sustain attention to negative pictures for 1000 ms and focus away from positive ones. The results showed that participants gazed at the positive images longer and disengaged from the negative images more quickly in the PT. However, there is no change in attentional processes in the NT. The two groups showed no difference in emotional responses and recovery from stress. This disengagement training was supposed to increase attentional bias towards positive information and facilitate disengagement from negative information, which has significant relevance for the treatment of depression. To investigate the relationship between attention bias and other influencing factors. Lu et al. (2017) proposed a free-viewing eye movement task to compare the differences in attentional bias between depressed patients and healthy subjects at different ages. The trials were divided into two groups: happy-neutral faces and sad-neutral faces. The participants were also divided into two groups: young (18–30 years old) and middle-aged (31–55 years old). Compared with healthy subjects, depressed patients tended to pay less attention to the positive stimuli and more to the negative stimuli. Among major depressive disorder (MDD) patients, the middle-aged group had less positive attention bias than the younger group, and there was no difference in negative attention bias between the two groups. This study further demonstrated that emotional bias in depression was correlated with age. The complex visual arrays were employed by Klawohn et al. (2020) to examine the attention bias of depressed patients to facial expressions. Participants were free to view two sets of four-by-four arrays of facial expressions (see Figures 2iii). The first group was made up of sad and neutral faces, and the other of happy and neutral faces. At the same time, Eyelink 1000 + recorded the dwell time of participants on different facial expressions. Compared to healthy individuals, depressed individuals spend more time gazing at sad faces, which indicates an abnormal attentional bias to negative stimuli. Moreover, healthy and depressed groups had higher dwell times on happy expressions. Besides, the study further demonstrated that attentional bias to negative stimuli was not associated with the severity and chronicity of depression but associated with external environmental factors, including childhood trauma and sad events in contemporary life.

It is evident that vision-based sensors can be utilized to assess attentional biases in mental diseases. Table 1 provides a good overview summary of examples of the use of vision-based sensors for mental disease diagnosis. People with AD and depression pay less attention to positive and new things. And negative information affect people with depression for a longer period. People with ASD lose sight of the whole and focus on the details. Deep learning was used to diagnose AD and ASD patients and achieved the highest accuracy rate of 92.31%. Gaze-based devices, i.e., eye trackers, have the advantages of being non-invasive and wearable, and eye-tracking data are easily collected. However, the presence of blink and jitter in eye movement data introduces noise and errors for diagnosis. In addition, this method has some limitations including long preparation time, fragility, and non-myopic participants.

**TABLE 1:** Examples of vision-based sensors towards mental disease diagnosis. SMMSE, mini-mental status examination; CPT, conners continuous performance test; AD, alzheimer’s diseases; LR, logistic regression; SVM, support vector machine; PT, positive training; NT, negative training; MDD, major depressive disorder.

Bio-sensors	Mental diseases	Equipment/Technology	Diagnostic standards	Assessment standards	Results	Advantages	Ref
Vision-Based sensors	Alzheimer’s diseases	Binocular eye-tracking system	The novelty preference for new and repeated images	SMMSE and CPT	AD patients have less novelty preference	Non-invasive, non-verbal	Chau et al. (2017)
	Autism spectrum disorder	Gazepoint GP3 video-based eye-tracker	Features of gaze and non-gaze from the web-related tasks	LR	75% accuracy	Large-scale detection and unobtrusive tasks	Yaneva et al. (2018)
		Tobii Pro Glasses 2	Features of visual fixation and session length	SVM	92.31% accuracy	Diagnosis through face-to-face conversations	Zhao et al. (2021)
	Depression	Tobii tx-120 eye-tracker system	Difficulties in Attentional disengagement	Time of disengaging attention	People with depression spend more time	Easy to assess data	Sanchez et al. (2013)
		The eye-tracking system by the iView 9 Hi-Speed system from SMI	Attentional bias in the PT and NT	Time of disengagement	People with high levels of depression have difficulty disengaging	Repetition training has therapeutic relevance	Ferrari et al. (2016)
		Tobii T120 Eye-tracker	Attentional bias in different facial expressions/Different age groups	Happiness and sadness bias scores	Middle-aged MDD patients have a low preference for positive faces	Proved that emotional bias is related to age	Lu et al. (2017)
		Eyelink 1000+	Attentional bias in sad-neutral and happy-neutral groups	Dwell-times	Patients dwell on sad things for a long time	Free-viewing technique	Klawohn et al. (2020)

### 3.2 EEG signal sensors

**FIGURE 3:**
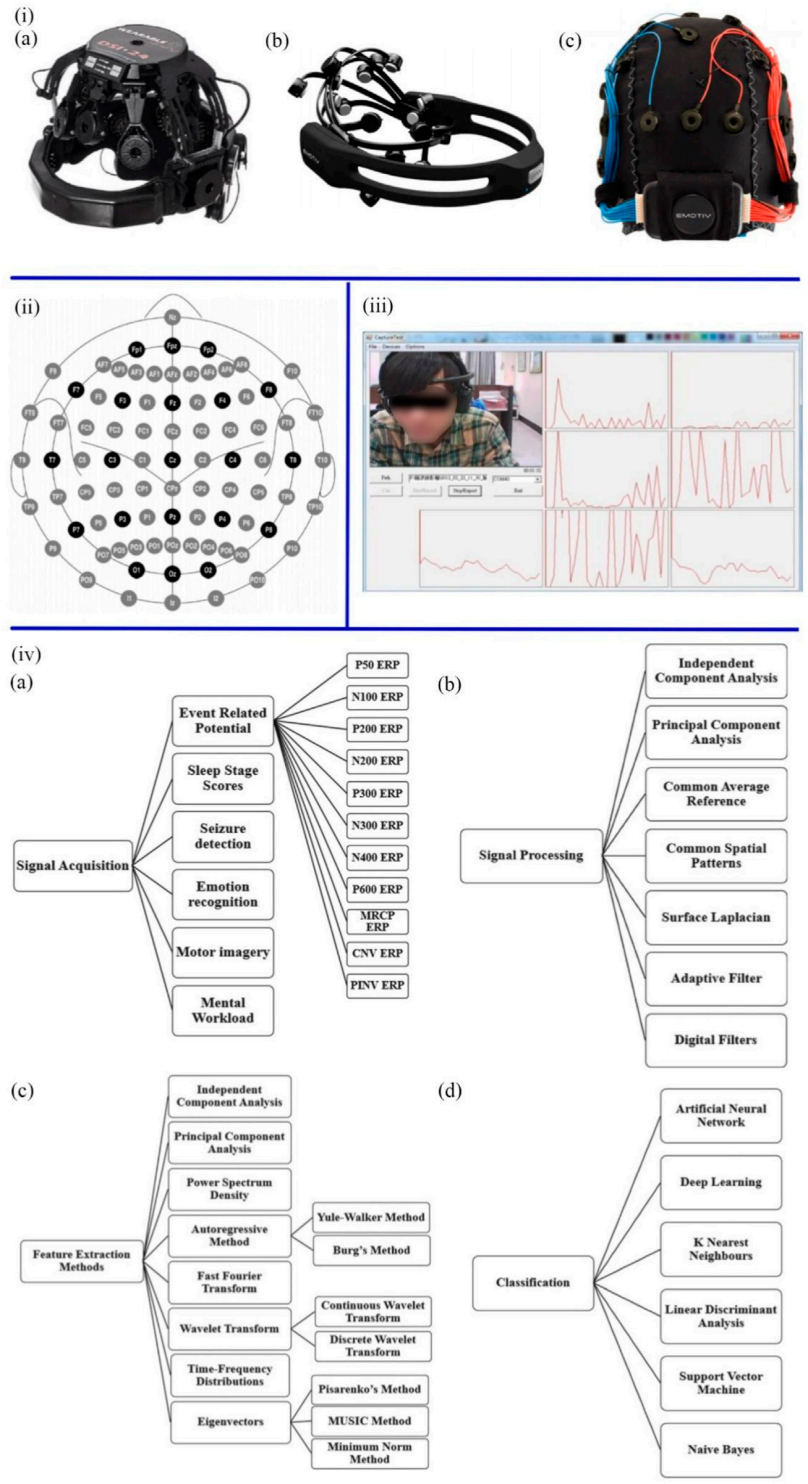
**(iA)** Dry EEG devices. **(B)** Saline solution EEG devices. **(C)** Soft gel-based EEG devices (Soufineyestani et al., 2020). **(ii)** The position and name of each electrode in the 10 ± 20 system (Soufineyestani et al., 2020). **(iii)** Real-time EEG signal recording system (Liu et al., 2013). **(ivA)** The methods of signal acquisition. **(B)** The types and techniques of signal processing. **(C)** Methods of feature extraction. **(D)** Machine learning algorithms (Sachadev et al., 2022).

Brain function is based on electrical signals among neurons in the brain. Due to the mood, mental state and attention being all controlled by different brain regions, mental diseases caused by brain damage can be diagnosed by EEG (Liu et al., 2013). EEG is a non-invasive, effective tool for measuring the electric activities of the brain and monitoring the changes in brain functions at rest or during stimulation. The principle is to measure tiny fluctuations in the electrical current between the skin and the sensor electrodes, amplify the current and perform filtering (Sullivan et al., 2007). The American clinical neurophysiology society recommends collecting brain electromagnetic activity through 10–20 and 10–10 electrode placement systems (Figures 3ii (Acharya et al., 2016). The capital letters F, C, T, P, and O stand for frontal, central, temporal, posterior, and occipital, respectively, and represent where the electrodes are attached to the skull. The numbers indicate the left and right sides of the brain (Abhang et al., 2016). When collecting EEG data, people should wear an EEG device to maintain a consistent electrical connection between the electrodes of the sensor and the scalp, which can be achieved by various methods (Figures 3i, iii), including dry EEG devices, saline solution EEG devices, and soft gel-based EEG devices (Soufineyestani et al., 2020). The dry EEG devices do not require any saline and gel, or even direct connection to the scalp, enabling a shorter up-front setup time than wet EEG devices. As computer-aided diagnosis (CAD) has become an important part of the medical industry, some researchers have attempted to diagnose mental diseases by using machine learning techniques to extract features from EEG signals (Khare and Bajaj, 2020; Khare et al., 2020).

As shown in Figures 3iv, collecting raw EEG signals involves many activities and methods, including Event-Related Potential, Emotion Recognition, Sleep Stage Scores, Motor Imagery, Seizure Detection Mental Workload. According to the frequency range of EEG signals, the central frequencies of human brain waves can be divided into Alpha, Beta, Delta, Gamma, and Theta bands (Liu et al., 2013). The methods of preprocessing include Adaptive Filter, Surface Laplacian, Independent Component Analysis, Common Spatial Patterns, Common Average Reference, Surface Laplacian, and Principal Component Analysis, which purpose is to improve the signal-to-noise ratio, remove artefacts, interference, and noise, and retain the pure EEG signal. After preprocessing the EEG data, various features are extracted. Wavelet Transform, Fast Fourier Transform, Principal Component Analysis, Independent Component Analysis, Power Spectrum Density, Autoregressive Method, Eigenvectors, and time-frequency Distribution can be utilized. Artificial intelligence and machine learning models, including K Nearest Neighbors, Support Vector Machine, Deep Learning, Artificial Neural Network, Linear Discriminant Analysis, and Naive Bayes, were used to calculate and analyze these features to classify healthy people and patients (Sachadev et al., 2022). Presently, EEG can diagnose AD, sleep disorders, and brain tumours (Cai et al., 2020; Duan et al., 2020; Akbari et al., 2021a). Recent studies have focused on detecting ASD, depression, epilepsy and schizophrenia.

Social communication is based mainly on nonverbal behavior to understand the intentions of others, e.g., gestures, actions, and emotions. These past studies provides evidence that people with ASD often have difficulty with social communication and recognition of facial emotions (Black et al., 2017; Tawhid et al., 2020). According to these symptoms, Paula et al. (2017) proposed an approach to diagnosing ASD by analyzing the changes in high-frequency EEG as children observe three different facial expressions (happy, neutral, and angry). In this visual stimulation task, photos with different expressions were played for 3 s each with intervals of 0.5–1.0 s controlled by Mangold Vision 3.9. The EEG-1200 measured EEG data about neural activity caused by the stimulus, and eye-Tech TM3 ensured that participants viewed fixed points and visual stimuli during this time. The EDF Browser converted each subject’s raw EEG data into ASCII format. Python programming language and MATLAB code were applied to process these EEG data. Their mean and standard deviation could be calculated to observe changes in local field potentials associated with events. After comparing EEG data from 8 children with ASD and 8 healthy children, the study found that the ASD group presented a more vital power spectrum in high frequencies (above 30 HZ) compared to controls in some regions, including the frontal, occipital, and mid-parietal regions of the brain. The EEG signals of the occipital and central parietal regions showed significant differences. The central region and parietal lobe presented similar patterns. Consequently, the findings demonstrated that the EEG activity of children with and without ASD showed different quantitative patterns of the power spectrum when observing facial expressions. Another effective diagnostic system was developed by Tawhid et al. (2020), which can automatically identify ASD based on a 2D spectrogram images of EEG signals. The novelty of the study is in the conversion of EEG signals into time-frequency based spectrogram images. The preprocessed EEG signal was converted into a two-dimensional (2D) image by short-time Fourier transform. Then used ternary CENTRIST to extract texture features. 10-fold cross-validation by the SVM classifier yielded an average classification accuracy (ACC) of 95.25%, a sensitivity of 97.07%, and a specificity of 90.95%.

**FIGURE 4:**
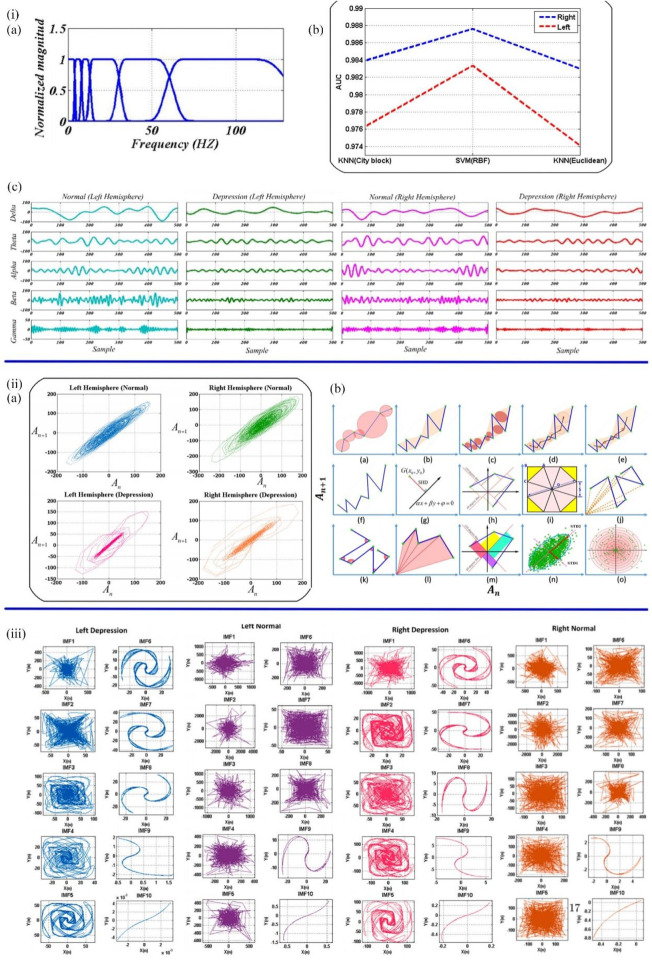
**(iA)** EWT filter bank. **(B)** The decomposed rhythms from the left and right hemispheres of the normal and depressed groups. **(C)** The AUC values of SVM and KNN classifiers (Akbari et al., 2021b). **(iiA)** The samples of normal and depressed RPS for the left and right hemispheres. **(B)** Quantified the geometric features of the RPS pattern of EEG signals in 2D space (Akbari et al., 2021a). **(iii)** 2D-IMFs of EEG signals in depression (Sadiq et al., 2021).

Currently, EEG is also a popular tool to investigate the presence of depression biomarkers. Previous studies have demonstrated that the EEG signals of depressive patients presented as unpredictable and have lower morphological complexity (Sharma et al., 2018; Sadiq et al., 2020). Sadiq et al. designed a user-friendly method based on centered correntropy (CC) of rhythms in empirical wavelet transform (EWT) to classify EEG signals in people with 22 depression and 22 non-depression subjects (Akbari et al., 2021b). The non-stationary EEG signals were decomposed into rhythms by EWT (Figures 4i,A), and the EWT filter bank was created. CC was calculated from the decomposed delta, theta, alpha, beta, and gamma rhythms, regarded as the discrimination feature, and then fed to SVM and KNN (K Nearest Neighbors) classifiers (Figures 4i,B, C). The area under the receiver operating characteristic curve (AUC) quantifies the ability to classify depression and standard EEG signals. The AUC values of the SVM classifier with radial basis function kernel and KNN classifier with city block and Euclidian were compared to prove the performance of the two classifiers. The proposed method ultimately achieved 98.76% ACC in a 10-fold cross-validation strategy. Subsequently, Sadiq et al. further employed reconstructed phase space (RPS) of EEG signals and geometrical features for depression detection (Akbari et al., 2021a). The method of classification and evaluation of the selected features was the same as the previous experiment, which used SVM and KNN classifier. The difference was in the processing stage. The EEG signals of the left and right hemispheres were plotted by PRS in 2D space (Figures 4ii), and 34 nonlinear geometrical features were extracted from these characteristics. Four optimization algorithms, Ant Colony Optimization (ACO), Grey Wolf Optimization (GWO), Genetic Algorithm (GA), and Particle Swarm Optimization (PSO), were used to reduce the feature vectors, and the performance was compared. GA achieved better performance due to a 58.8% reduction in feature vector arrays. The framework using the PSO algorithm and SVM classifier finally achieved 99.3% ACC and a Matthews correlation coefficient (MCC) of 0.98 in the right and 0.95 in the left hemispheres. Therefore, it could be concluded that EEG signals can be used as biomarkers to detect depression. Furthermore, EEG signals from the right hemisphere are more critical for detecting depression than those from the left. In the following work, Sadiq et al. (2021) found a novel CAD system to detect depression automatically. In this study, bipolar channels “FP1-T3" and “FP2-T4" from the left and right brain were used to collect EEG records for 10 min at 256 Hz sampling frequency. By new 2D modelling of intrinsic mode functions (2D-IMFs) (Figures 4iii), Binary Particle Swarm Optimization (B-PSO) algorithm, and KNN classifier, depression and epilepsy could be classified and diagnosed. As a result, this system possessed various advantages, including time-saving, a high classification accuracy of 93.35%, and multirole adaptability. This work provided a novel way to diagnose two mental diseases with one algorithm.

Another EEG signal classification method for the diagnosis of epilepsy was proposed by Akbari and Esmaili (2020). The novelty of this approach lies in the use of second-order difference maps and phase-space reconstruction to map EEG signals in 2D space. 500 interictal, ictal and normal EEG signals were extracted with 6 features in different aspects of distance in Cartesian space. These features are named circle area, area of the octagon, summation of vectors length, ircular radius in triangles, triangle area and centroid to centroid, respectively. More regular geometries appear in 2D projections of interictal and normal EEG signals. At the same time, the edges of the 2D EEG projection signals in the ictal group appeared clearer than those in the other two groups. This method achieves 99.3% ACC under the 10-fold cross-validation strategy through SVM and KNN classifiers. The same method was also applied to the diagnosis of schizophrenia by Akbari et al. (2021c). EEG samples included 14 subjects with schizophrenia and 14 controls. Recorded on a 10–20 system with 19 EEG channels. Fifteen graph features were extracted to evaluate the chaotic behavior of phase space dynamics. The results showed that the KNN classifier with City-block distance achieved 94.80% ACC under the 10-fold cross-validation strategy. Another machine learning classifier for diagnosing schizophrenia based on EEG signals of event-related potentials (ERP) was designed by Zhang (2019) The EEG signal of motor actions was shown to be one of the biomarkers for identifying schizophrenia (Ford et al., 2014). Hence, the characteristics of the EEG signals of 49 schizophrenia patients and 32 healthy controls were captured by the sensory tasks including button pressing or/and auditory. The results proved that the highest classification accuracy of 81.1% was achieved by the RF algorithm.

The different diagnostic methods based on EEG signals are shown in Table 2. Greater activation in the frontal, occipital, parietal, and central regions could be considered diagnostic criteria for ASD. CADS (i.e., Fast Fourier Transform, Wavelet Transform) were utilized to reduce the complexity of EEG signals. And automatic classification was realized by machine learning methods (i.e., SVM, KNN, Ternary CENTRIST and RF) for extracting features of EEG signals. The classification accuracies for both depression and epilepsy were as high as 99.3%. And the diagnostic accuracy rate of schizophrenia reached 94.8%. Furthermore, the goal of simultaneous diagnosis of two mental diseases (epilepsy and depression) was achieved by EEG. The experiments in Table 2 are all based on short-range EEG. Although this method is relatively quick and low cost, there are limitations in the ability to capture the abnormal brain waves of patients during the onset. In the future, 24-h monitoring and mobile brain-computer interface (BCI) will gradually become potential directions for general EEG applications (Chi et al., 2012; Amaral et al., 2018).

**TABLE 2:** Examples of EEG signal sensors towards mental diseases diagnosis. ERP: event related potential; ASD, autism spectrum disorder; Ternary CENTRIST, a texture classifier; CC, correntropy; ACC, average classification accuracy; KNN, k nearest neighbors; EWT, empirical wavelet transforms; B-PSO, binary particle swarm optimization; RF, random forest.

Bio-sensors	Mental diseases	Equipment/Technology	Diagnostic standards	Assessment standards	Results	Advantages	Ref
EEG signal sensors	Autism spectrum disorder	EEG-1200 and ERP	Attentional bias in angry, happy, and neutral faces	High-frequency (above 30 HZ) EEG variations	ASD group has strong activation in the center-parietal, occipital and frontal area	Showed quantitative differences in two groups	Paula et al. (2017)
		A g.tec EEG cap with Ag/AgCl electrodes	Time-frequency based spectrogram images	Ternary CENTRIST and SVM	95.25% ACC	High accuracy and sensitivity for ASD diagnosis	Tawhid et al. (2020)
	Depression	Recorded from both hemispheres by a bipolar montage	The CC of the rhythms decomposed by EWT	SVM and KNN	99.05%, 98.47%, and 98.76% ACC	Reduced pre-processing, rhythm extraction, and algorithm complexity	Akbari et al. (2021b)
		Bipolar channels (FP1-T3 and FP2-T4 channels)	34 geometrical features were extracted from RPS	SVM and KNN	99.3% ACC	Fast and inexpensive, high classification accuracy	Akbari et al. (2021a)
			The significant features were selected by B-PSO	KNN	93.35% ACC	Diagnostic two mental diseases by one unified algorithm	Sadiq et al. (2021)
	Epilepsy	500 EEG signals from Bonn university database	Features were extracted on different aspects of distance in Cartesian space	SVM and KNN	99.3% ACC	High accuracy, sensitivity and specificity	Akbari and Esmaili (2020)
	Schizophrenia	10–20 EEG montage with 19 EEG channels		KNN	94.80% ACC	Fully automatic and inexpensive	Akbari et al. (2021c)
		ERP EEG signals with nine selected electrodes	Three sensory tasks	RF	81.1%	Fast, simple and effective	Zhang (2019)

### 3.3 EOG signal sensors

**FIGURE 5:**
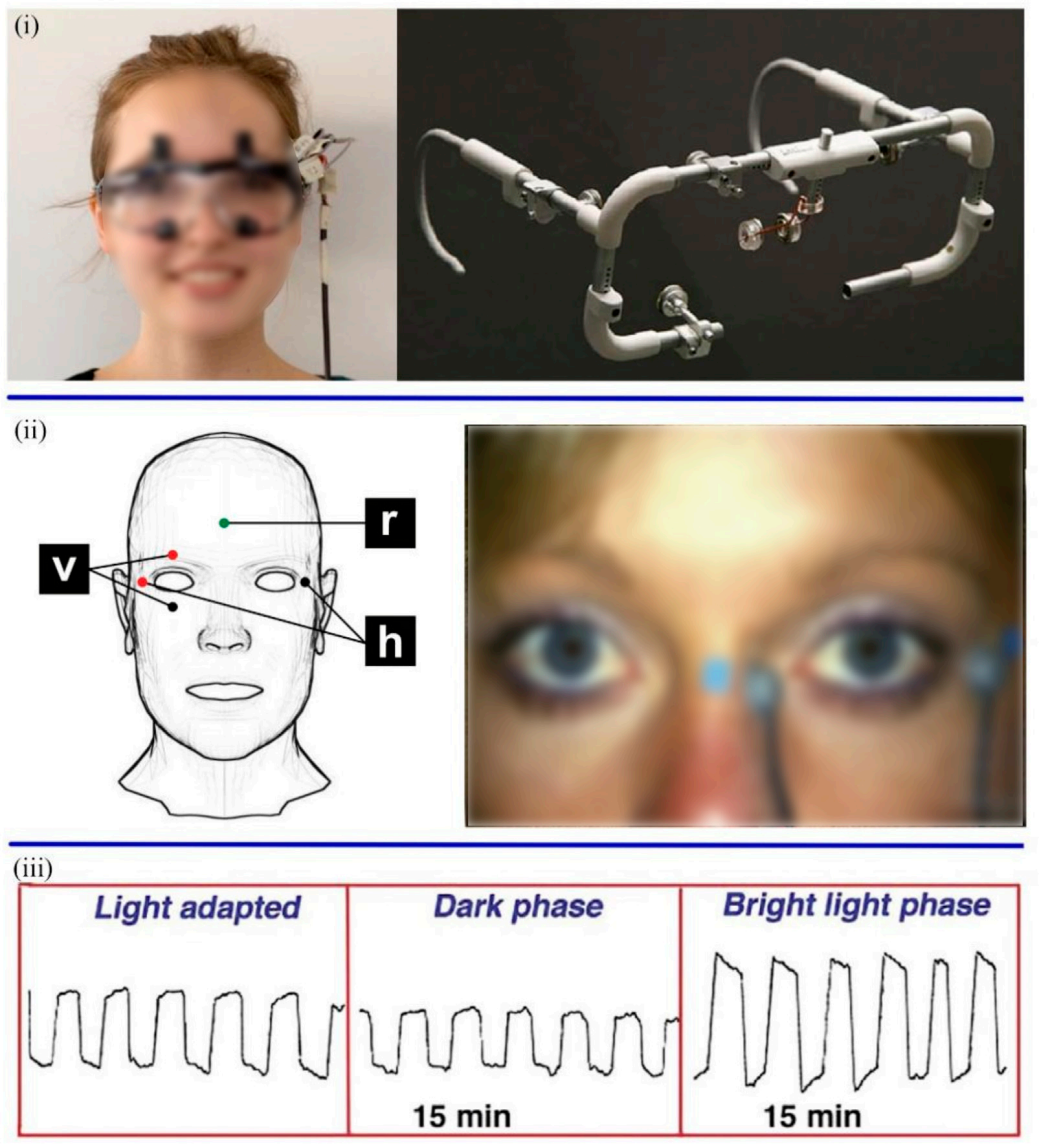
**(i)** The wearable EOG equipment (Majaranta and Bulling, 2014). **(ii)** Position of electrode for recording EOG signal (v: vertical, h: horizontal, and r: reference) (Bulling et al., 2011; Creel, 2019). **(iii)** EOG eye movements were recorded in three phases (Creel, 2019).

The electrooculogram sensor is another tool that can detect mental diseases. Both EOG and EEG use an array of electrodes to capture signals (Kaur, 2021). EOG is a method of sensing eye movement that measures the retinal electrostatic potential between the retinal pigment epithelium and photoreceptor cells. EOG signals are recorded by placing a series of skin electrodes on the lateral and medial canthus (or above and below the eyelids) of each eye, which measure horizontal (or vertical) eye movements. Moreover, ground electrodes are attached to the earlobe or forehead (Figure 5) (Bulling et al., 2011; Creel, 2019). Measuring EOG signals requires patients to acclimatize in a well-lit room for at least 30 min. Before the test, there was a light acclimation period of about 10 min. After the electrodes are attached, there are 15 min of dark and bright light phases. The movement of the eyes creates a voltage fluctuation of approximately 2–5 mV between the electrodes on either side of the eye, which is plotted on the computer (Creel, 2019).

The abnormality of smooth pursuit eye movement (SPEM) was demonstrated as a genetic marker of schizophrenia in preliminary experiments (Siever et al., 1985; Matsue et al., 1994). Moreover, the same dysfunction is present in first-degree relatives of schizophrenics (Holzman et al., 1974). Kathmann et al. (2003) described a method to evaluate the integrity of the SPEM system by comparing the eye velocity and target velocity. The study subjects included 103 schizophrenic patients, 53 relatives of patients and 72 healthy controls. In the eye-tracking task, the movement of white circles was tracked by subjects, which was combined with auditory and visual distraction tasks. At the same time, horizontal eye movements were recorded by EOG. The pursuit gain, the velocity of eye movement divided by the velocity of target movement, showed noteworthy gain deficits in both schizophrenia and affective disorders. And unaffected biological relatives of schizophrenia patients had lower pursuit gain than healthy subjects. This finding further supported that deficit in the gain of SPEM could be regarded as a phenotypic marker of genetic predisposition to schizophrenia. In addition to the abnormity of SPEM, Quality Extinction Test (QET) is another indicator that can identify the damage to the parietal and frontal lobes (Scarone et al., 1982). Scarone et al. reported a higher incidence of left-side extinctions on QET in patients with schizophrenia (Scarone et al., 1982; Scarone et al., 1983). Accordingly, this research team further performed a method to simultaneously investigate two features of central nervous system disorder in schizophrenia, namely abnormalities of SPEM and abnormalities of QET (represented by loss of touch). The results of the experiment indicated that more patients with left-side extinctions had SPEM abnormalities, which suggested that simultaneous impairment of these two psychophysiological indicators can be applied to detect schizophrenics (Scarone et al., 1987). Besides, Lencer et al. (2000) tried to determine whether genetic factors influenced families with sporadic schizophrenia (single occurrences of schizophrenia) by dysfunction of smooth pursuit performance. The saccade amplitudes, saccade rates, and gains of three kinds of family including 8 families with sporadic schizophrenia (n = 44), 8 families with multiple occurrences of the disease (n = 66), and 9 healthy families (n = 77) were recorded by infrared eye-tracker. The amplitudes and rates of saccades and gain values in families with sporadic schizophrenia significantly differed from healthy controls at 30°/sec triangle-wave stimulation, but not from familial-schizophrenia families. In addition, target direction significantly affected smooth-pursuit maintenance in families with familial and sporadic schizophrenia. The results supported the hypothesis that genetic factors may be indicated in sporadic-schizophrenia families.

Due to the development of deep learning, research on the automatic detection of sleep disorders through EOG has been gradually carried out. Rahman et al. (2018) developed a dynamic and automatic sleep scoring system based on single-channel EOG. In this work, EOG signals of 38 patients in different stages of sleep, namely Awake, S1, S2, S3, S4, and REM (rapid eye movement) (Rechtschaffen AJBis, 1968), were extracted from three public databases (SLEEP-EDF, SLEEP-EDFX, and ISRUC-SLEEP). And these signals were decomposed by Discrete Wavelet Transform (DWT) in order to extract highly discriminatory features from them, e.g., Moment-based Measures. After that, Neighborhood Component Analysis, a feature reduction technique, was employed to reduce features and compact the model. RF, SVM, and RUSBoot (Random Under-Sampling Boosting) algorithms were used to classify the data from the three databases in different classification stages, respectively. The proposed method had higher accuracy compared with other sleep stage classification techniques based on single-channel EEG, single-channel EOG, and dual-channel EOG, and made significant progress in the S1 sleep stage, which is difficult to detect visually. Besides, Tasnim et al. (2019) designed another sleep state classification method based on single-channel EOG. The raw data were obtained from the SLEEP-EDF database, which was divided into 6 Sleep stages as above. The features of EOG signals were extracted and reduced through Variational Mode Decomposition and Neighborhood Component Analysis to obtain meaningful statistical features like Spectral Entropy Measures. In the training model stage, three widely used classifiers, RUSBoost, RF, and KNN, were used. It has been found that the proposed algorithm achieves 96.537%, 93.05%, 90.57%, 89.21%, and 88.083% overall accuracy in the classification stage of from 2-class to 6-class sleep, respectively, and 65.092% accuracy in the S1. In the work of Sharma et al. (2022), a system for the automatic recognition of sleep disorders based on a single-modal EOG signal was proposed. Besides, the EMG signal was also recorded. The database used in the study was the PhysioNet database, which contained the records of 16 healthy patients and 92 patients with sleep disorders. DWT was used to decompose EOG and EMG signals and extract Hjorth parameters (HOP) features. Highly discriminative HOP features were sent to different classifiers including SVM, Boosted Tree (BT), and Ensemble Bagged Tree Classification (EBTC). Among them, the EBTC classifier with 10-fold cross-validation technology showed the best performance, achieving 94.3% accuracy when using the deep sleep stage data.

Schizophrenia and sleep disorders can be diagnosed based on the EOG signal. As shown in Table 3. Schizophrenia patients and their unaffected relatives showed significantly lower pursuit gain and left-sided patients showed abnormal SPEM. In addition, machine learning methods including RUSBoost, EBTC, RF, SVM and KNN were used to extract the features of eye signals during sleep, and the highest accuracy rate reached 96.537%. EOG devices are lightweight, easy to wear, and can even capture eye signals during sleep. However, a significant amount of time is required for pre-examination preparation and pre-adaptation before the examination.

**TABLE 3:** Examples of EOG signal sensors towards mental diseases diagnosis. SPEM, smooth pursuit eye movement; QET, quality extinction test; RF, random forest; RUSBoot, random under-sampling boosting; BT, boosted tree; EBTC, ensemble bagged tree classification; REM, rapid eye movement.

Bio-sensors	Mental diseases	Equipment/Technology	Diagnostic standards	Assessment standards	Results	Advantages	Ref
EOG signal sensors	Schizophrenia	Silver-silver chloride electrodes	Intactness	Pursuit gain	Low pursuit gains in patients and their unaffected relatives	The family specificity of schizophrenia was further verified	Kathmann et al. (2003)
			Eye movement and neuropsychological tests	SPEM and QET characteristics	Abnormal SPEMs were more present in left-side extinguishers	Investigated simultaneously two indices	Scarone et al. (1987)
		High-resolution infrared reflection oculography	Eye-tracking dysfunction	Gains, rates, and amplitudes of saccades	Genetic factors exist in families with schizophrenia	Further precisely define the schizophrenia phenotype	Lencer et al. (2000)
	Sleep disorder	ISRUC-SLEEP, SLEEP-EDFX, and SLEEP-EDF databases	Awake, S1, S2, S3, S4 and REM	SVM, RF, and RUSBoot	Superior accuracy in RUSBoost	Low cost	Rahman et al. (2018)
		Sleep-EDF database		RUSBoost, RF, and KNN	88.083%–96.537% overall accuracies and 65.092% accuracy in S1	Significantly improved accuracy and efficiency	Tasnim et al. (2019)
		PhysioNet database	Awake, N1, N2, N3 and REM	SVM, BT, and EBTC	94.3%	Portable and automated device	Sharma et al. (2022)

### 3.4 VR-based sensors

**FIGURE 6:**
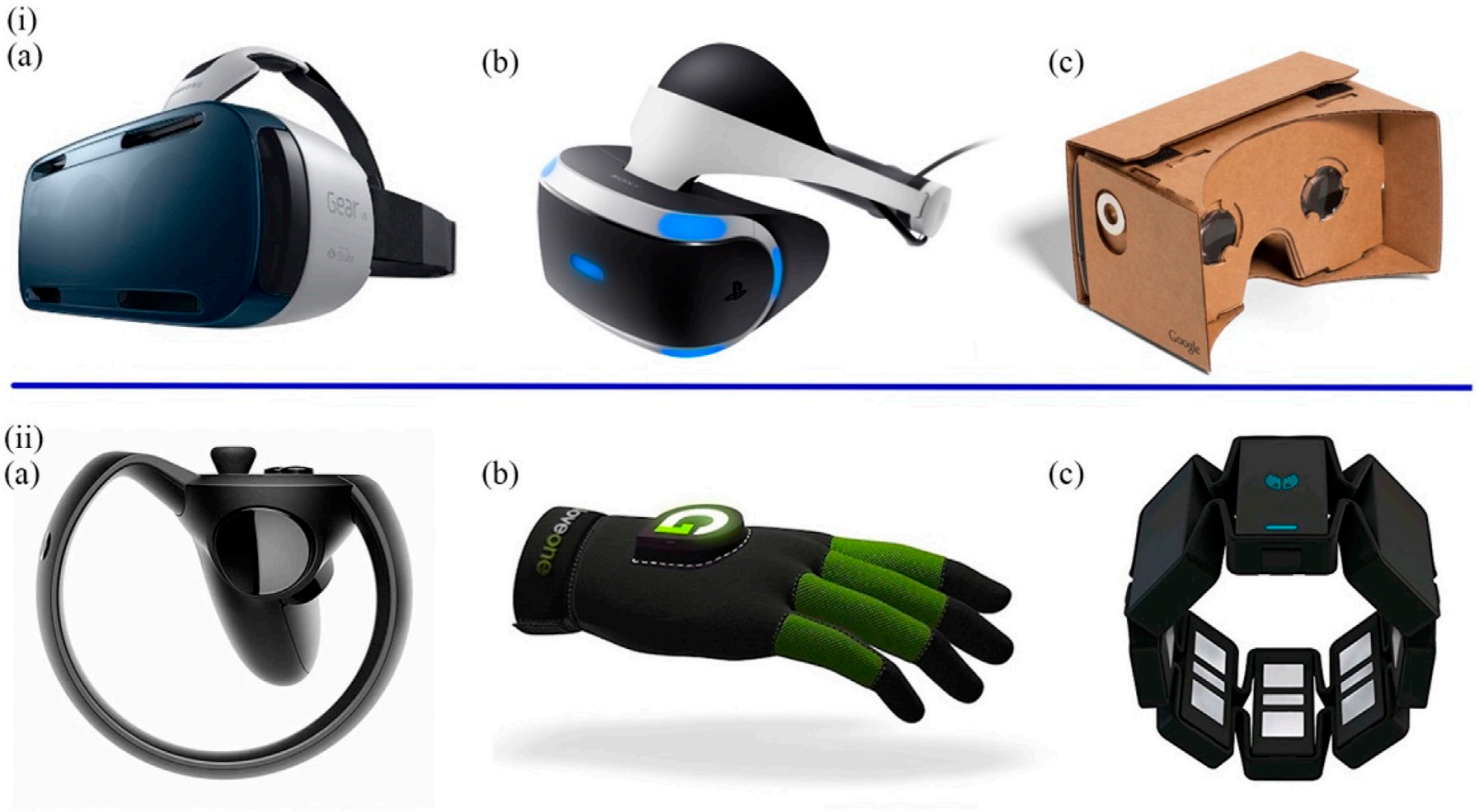
**(i)** The most prominent HMDs. **(A)** The Samsung GearVRInnovator Edition. **(B)** The Sony PlayStation VR. **(C)** The Google Cardboard. **(ii)** The most typical controllers. **(A)** The Oculus Half Moon. **(B)** The Glove ONE. **(C)** The Myo gesture control armband (Anthes et al., 2016).

Emerging VR applications, which are intended for navigation and orientation, cognitive and memory functions, facial identification, and other instrumental activities of daily life, have exhibited practical uses in neuropsychological assessments (García-Betances et al., 2015). Compared with the three methods mentioned in Section 3.1 and Section 3.3, more kinds of mental disease can be diagnosed through VR. VR transmits information from real life to the virtual world, providing the possibility for people to perform activities in the virtual space (Goharinejad et al., 2022). From a research perspective, the use of VR allows for the repetition of clinical practice and continuous data collection in a virtual world (Tieri et al., 2018), and provides excellent visual and auditory immersion and interaction during tasks (Climent et al., 2021). According to different degrees of immersion, VR can be divided into fully immersive, semi-immersive and non-immersive. Fully immersive VR devices are typically equipped with head-mounted displays (HMDs), data gloves, gesture control armbands, gamepads, and speakers (Figure 6), placing participants inside a virtual environment for the highest level of immersion. Semi-immersive VR presents a visual virtual environment through a relatively large flat-screen display. In non-immersive systems, participants interact using traditional PC monitors, keyboards, and mice (Kim et al., 2009; Anthes et al., 2016; Mehrfard et al., 2019; Yeung et al., 2021).

In the early stages, numerous researchers used VR to diagnose the impairment of daily activities in AD. Allain et al. (2014) assessed daily mobility impairment in AD patients through a non-immersive virtual coffee task. Performance evaluation includes five indicators: Time to completion, accomplishment score, omission errors score, commission errors score and total errors. The results showed that AD patients fared worse than healthy controls. Yamaguchi et al. (2012) developed a dual-modality VR platform for training AD patients on toast and coffee tasks. AD patients had more omissions, mistakes, and repetitive behaviors during tasks than controls, demonstrating deficits in daily activities in AD patients. However, AD patients achieved performance levels similar to controls in a short period of time after learning. In recent years, increasing evidence has shown that impaired spatial navigation and orientation deficits are important biomarkers for cognitive assessment in patients with MCI. Patients exhibited impairments in the ability to use egocentric (eye/head/body-based) and non-centric (map-based) navigational strategies, which were associated with impaired memory and decreased attention early in AD (Coughlan et al., 2018a; Coughlan et al., 2018b; Puthusseryppady et al., 2020). Serino et al. (2015) designed a non-immersive VR-based procedure to assess the ability for encoding, storing, and synchronizing different spatial representations. Participants included 15 amnestic MCI patients, 15 AD patients, and 15 healthy people as controls. The task required them to memorize the location of the plant and be able to find it later from other directions (the plant location was not marked). The results of the experiment suggested that patients with amnestic MCI have deficits in the ability to encode and store non-central viewpoint-independent representations. AD patients have specific deficits in storing non-central viewpoint-independent representations and synchronizing them with non-central viewpoint-related representations. Plancher et al. (2012) assessed central and episodic memory of participants based on a virtual active exploration task (as a car driver) and a passive exploration task (as a passenger). The results verified that spatial non-central memory could diagnose amnestic MCI patients.

**FIGURE 7:**
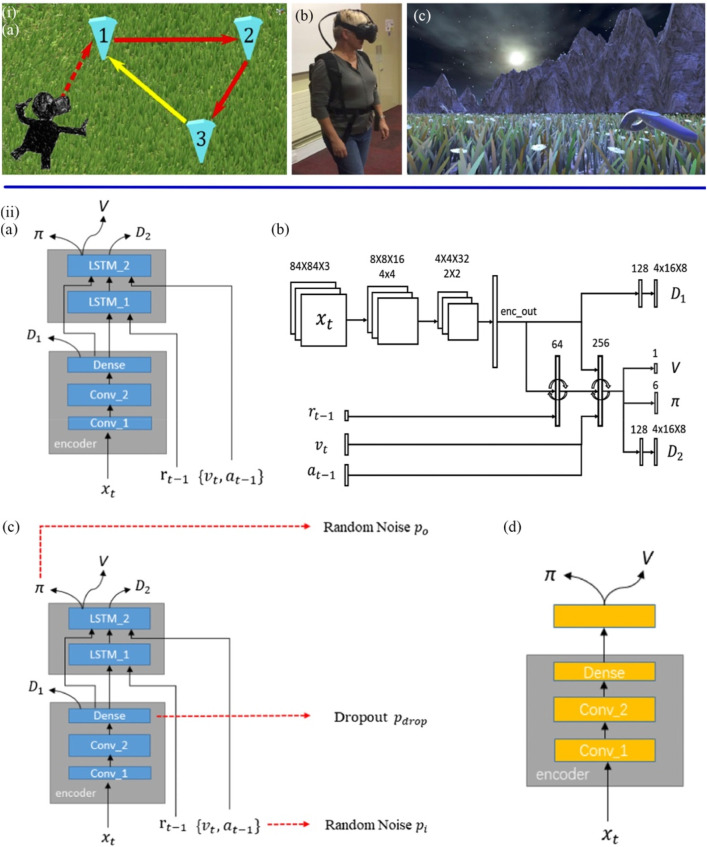
**(iA)** Participants were asked to walk to positions 1, 2, and 3 (marked) in sequence during the VR task and return to the unmarked 1 by memory. **(B)** Participants wear VR devices. **(C)** The participant tried to go back to 1 without the marker (Howett et al., 2019). **(iiA)** The based A3C navigation network structure (GA3C_LSTM) were used to simulate healthy individuals with normal cognitive levels. **(B)** Details on the size of each layer. **(C)** The Noise Navigation Network (GA3C_Noise) Models Cognitively Impaired MCI Patients. **(D)** The Dememory Navigation Network (GA3C_FF) simulates dementia patients with partial and complete loss of short-term memory (Jiang et al., 2020).

The emergence of advance VR equipment and technology allowed for a type of spatial immersion termed “telepresence". Howett et al. examined differences in entorhinal cortex-based navigation (Figures 7i) in 45 MCI patients *versus* 44 healthy controls based on immersive VR integration testing (Howett et al., 2019). Navigation performance correlated with MRI measurements of entorhinal cortex volume. Classification accuracy on a path integration task was compared with cognitive tests in early AD. Performance on the task was evaluated using three outcome metrics, including absolute distance error, scaled angle error, and scaled linear error. Statistical analysis used linear mixed effects models. It turned out that the MCI group showed significantly more errors in the navigation task. Furthermore, the pathway integration task demonstrated higher diagnostic sensitivity and specificity in diagnosing AD patients than the best cognitive tests. Puthusseryppady et al. (2022) further correlated performance on a VR navigation test with performance on community navigation to see if spatial disorientation could be predicted. The tests were divided into three parts. The Virtual Supermarket Test (VST) was a spatial navigation test on an iPad that assesses egocentric orientation, concentric orientation, and heading. Sea Hero Quest (SHQ) was a mobile game that measures the ability to navigate space. The Detour Navigation Test (DNT) was a round-trip route test based on a highly familiar environment. Participants will use egocentric and non-centric navigation strategies during the round trip. Compared with controls, AD patients showed impairments in the VST, SHQ, and DNT. The experiment also reflected the future VR-based diagnostic technology will be applied in everyday electronic products. With the popularity of computerization, the combination of wearable devices, deep learning and VR can provide a low-cost, automated way for disease screening and prediction (Tarnanas et al., 2014). Jiang et al. (2020) developed a 3D maze procedure to assess and train the navigation and cognitive abilities of AD patients and healthy individuals. The program was based on the asynchronous advantage actor-critic algorithm (A3C) to train the agent to simulate the cognitive degradation process of AD patients. And combine the neural network with the pathogenesis of AD patients to reveal the underlying mechanism leading to AD. As shown in Figures 7ii, behavior data in navigation was collected and analyzed through three models. Results showed that patient-mimicking navigation models were inferior to those representing healthy individuals in terms of average number of steps, path efficiency, and decision-evaluation ability.

In addition to AD, depression (Gould et al., 2007; Voinescu et al., 2023) and epilepsy (Grewe et al., 2014) can also be diagnosed by VR-based methods through spatial memory and navigation ability. Voinescu et al. (2023) assessed impairment of attention and inhibition in depressed patients via the Nesplora virtual aquarium. Participants were required to pay attention to and respond to the correct stimulus (fish) during the 18-min task. The stimuli included auditory and visual, with the aim of exploring the relationship of attentional deficits to specific sensory processing. The results demonstrated that the depressed patients had omissions and errors in the visual stimuli, but not in the auditory stimuli. And the outcomes of Nesplora aquarium were found to be highly correlated with those of standardized neuropsychological measures. In contrast to the diagnostic approach above, ASD (Cai et al., 2013; Kim et al., 2015) and schizophrenia (Freeman, 2008; Dyck et al., 2010) were diagnosed through VR tasks based on social impairment and emotional blunting. In the experiment by Kim et al. (2015), 42 participants (19 ASD children and 23 healthy controls) used a joystick to adjust the social distance from a virtual object while trying to identify different emotions expressed by the virtual avatar. The VR emotion sensitivity test (V-REST) (Kim et al., 2010) included six emotions, avatars of male and female gender, and four levels of emotional intensity. The observation was that both the ASD children and the control group showed relatively high accuracy in identifying happy emotions. On the other hand, interpersonal distance can be affected by social anxiety (March et al., 1997). Therefore, participants were instructed to use a joystick to adjust the distance to the avatar. Compared with controls, ASD patients showed less tendency to move towards avatars of happy emotions and to a greater extent away from avatars of negative emotions. However, most of the researches on diagnosing ASD, depression, schizophrenia, and epilepsy through VR technology were published around a decade ago (Dechsling et al., 2021). In recent years, the direction of research combining VR and mental diseases has shifted from diagnosis to treatment. Multiple research projects have reported the efficacy of VR-based training (Herrero and Lorenzo, 2020; Dellazizzo et al., 2021; Yen and Chiu, 2021). In addition, augmented reality (AR), as a relatively new concept, provides an effective experimental basis for further understanding and treatment of mental diseases (Lorenzo et al., 2019; Rohrbach et al., 2019).

In essence, VR-based sensors create countless possibilities for immersing participants in virtual tasks, such as navigation, social, and daily activity-related tasks as illustrated in Table 4. Through these virtual tasks, schizophrenia, epilepsy, depression, ASD, and AD can be distinguished from healthy controls. In the task, the computer application can modify the simulated environment according to the responses and actions of participants (Riva et al., 2020). Hence, the interaction with the virtual object can be repeated and adjusted countless times. However, creating engaging VR experiences is a highly complex challenge. The interaction of mainstream VR devices is currently based on handles, which requires participants to invest a certain amount of learning costs. The degree of cooperation of the participants can affect the results of experiment. Overly complex tasks can frustrate participants. Therefore, it is necessary to provide a more convenient spatial interaction mode to control virtual objects through eye control, voice and gestures, etc. In addition, older participants, especially those with AD, were more likely to feel dizzy and nauseated when using VR. Technical limitations and a failure to comprehend user perception and interaction during the design process are the reasons behind these poor experiences (Jerald, 2015). Perhaps blurring the boundaries between VR and AR and allowing users to adjust the degree of virtual and real can lead to a more ideal experience.

**TABLE 4:** Examples of virtual-environment-based sensors towards mental diseases diagnosis. VST, virtual supermarket test. SHQ, sea hero quest. DNT, detour navigation test. A3C, asynchronous advantage actor-critic. GA3C_Noise and GA3C_FF models, models simulating mild cognitive impairment and Alzheimer’s patients, respectively.

Bio-sensors	Mental diseases	Equipment/Technology	Diagnostic standards	Assessment standards	Results	Advantages	Ref
VR-based sensors	Alzheimer’s diseases and Mild cognitive impairment	17-inch laptop and mouse	Manipulation in coffee task	Time to completion, accomplishment score, commission errors score, omission errors score and total errors	Patients did worse than the control group	Initial support for the utility of VR task	Allain et al. (2014)
		Notebook PC, mouse, and headset	Manipulation in toast task and coffee task	Total time to complete tasks, total number of actions including omissions, and commission errors	Patients performed worse, but after learning they could perform similarly to controls	Effective training platform	Yamaguchi et al. (2012)
		ACER ASPIRE portable computer and Logitech Rumble F510 gamepad	Found the location of unmarked plants	Abilities to encode, store and synchronize different spatial representations	MCI patients had deficits in the abilities	Provided initial insights into the cognitive basis of patients	Serino et al. (2015)
		PC laptop computer	Tasks of virtual driving and riding in cars	Center information, context information and quality of binding	Patients had worse spatial memory scores	The damage of heterocentric spatial memory can be used as an early diagnostic clue	Plancher et al. (2012)
		HTC Vive iVR kit and 32 channel Siemens 3 T Prisma scanners	Absolute distance error	Absolute distance error, proportional angular error, and proportional linear error	Patients made more errors in navigation	Higher diagnostic sensitivity and specificity	Howett et al. (2019)
		IPad and mobile phone	VR navigation (VST and SHQ) and community navigation (DNT)	Non-centric and egocentric navigation strategies	patients showed impairments in navigation tasks	Games could be disease screening tools	Puthusseryppady et al. (2022)
			Navigation tasks in a 3D maze	A3C algorithm	GA3C_Noise and GA3C_FF models perform worse in navigation	Helped patients long-term assess their navigational abilities	Jiang et al. (2020)
	Autism spectrum disorder	320-degree spherical 3D screen	The task of communicating with virtual dolphins through hand gestures	Social behavior	Female patients refused to interact with dolphins more than male	Encourage patients to interact with dolphins in novelty ways	Cai et al. (2013)
		A Pentium PC, LCD monitor and joystick	VR emotion sensitivity test	Interpersonal distance and accuracy of emotional recognition	Patients showed less tendency to move toward the avatar of a happy mood	Integrated multiple emotional expressions (face, gesture, voice) and social distance	Kim et al. (2015)
	Depression		Virtual town navigation task and traditional spatial memory task	Performance in two tasks	Patients found fewer locations during navigation tasks	Proved that hippocampus related spatial memory deficits can be used as biological indicators	Gould et al. (2007)
		Galaxy S7 smartphone and Gear VR headset	Nesplora aquarium attention test	The reaction of visual and auditory stimuli	Effectively predicted symptoms of depression and anxiety	Included visual and auditory attention	Voinescu et al. (2023)
	Schizophrenia		Emotions on natural and virtual faces	Whether the patient has trouble recognizing emotions	The patients had obstacle in emotional recognition of the emotions expressed by natural characters and virtual characters.	The animation and parameters of the avatar can be easily adjusted	Dyck et al. (2010)
	Epilepsy		The task of space navigation in virtual supermarket	Quantity of products purchased correctly, and time spent	Patients showed significant impairment in learning and purchasing	The eight-day test provided more comprehensive information	Grewe et al. (2014)

### 3.5 Multiple-signal sensors

In recent years, studies have made significant progress in detecting mental diseases using three biosensors including vision-based, EEG signal, and EOG signal sensors. In addition to the single use of these biosensors, some researchers combine them to achieve higher detection accuracy and efficiency. Multi-signal sensors collect data in the same manner as the sensors described above alone. Eye movement data, EEG and EOG signals were collected through multiple tasks, including dynamic visual tasks, EEG and EEG recording tasks. The features of multiple signals are extracted and classified by machine learning algorithms for higher accuracy.

**FIGURE 8:**
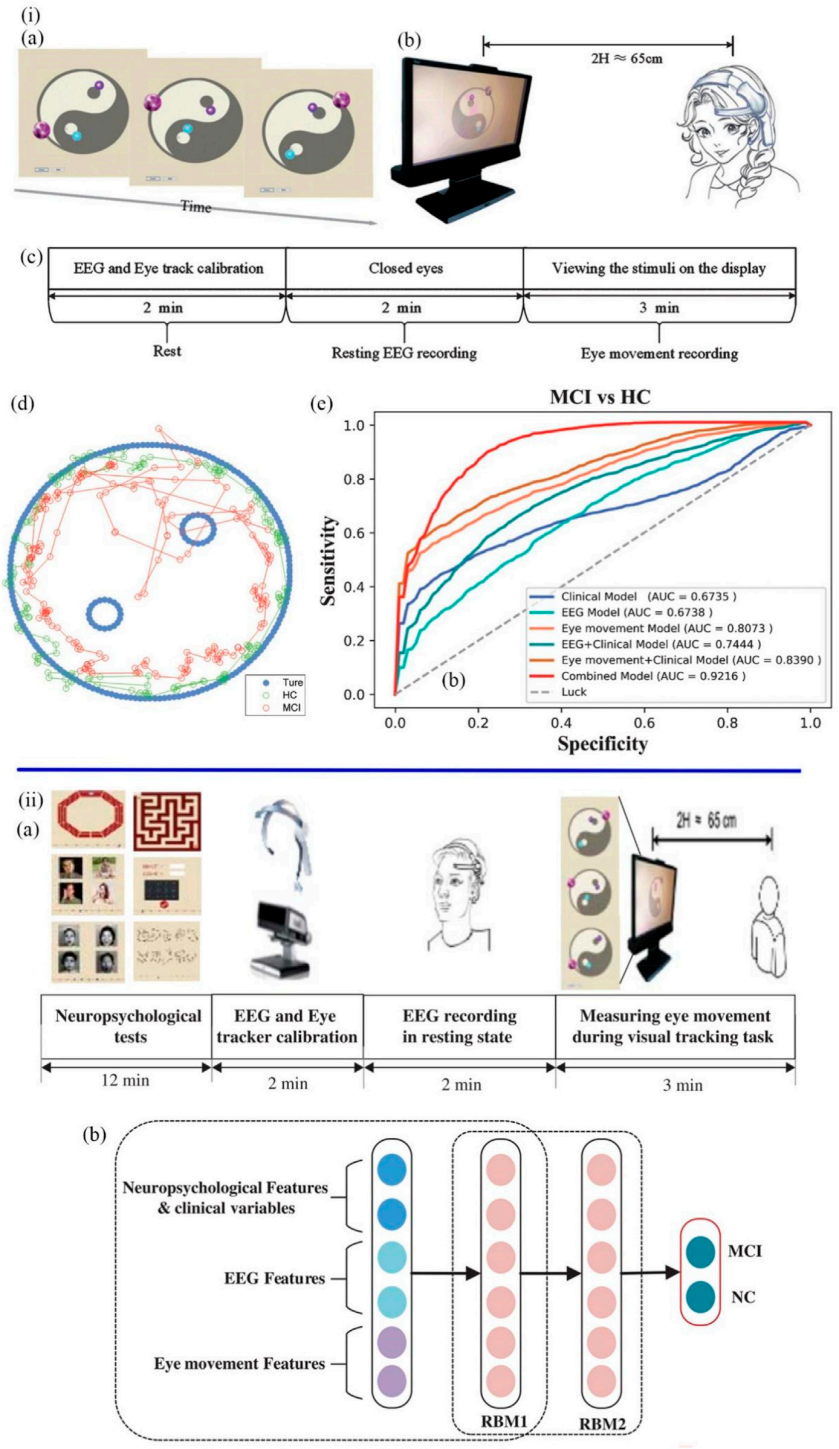
**(iA)** Visual stimulus task. **(B)** The viewing distance. **(C)** The experimental procedures for the visual tracking task consisted of 2 min of EEG recording and 3 min of eye movement recording. **(D)** The blue dots represent the movement of the task ball. The green dots and the red dots represent the locus of fixation distribution in the healthy MCI group, respectively. **(E)** The AUC of various MCI detection models (Jiang et al., 2019a). **(iiA)** The experimental procedures consisted of 12 min of testing, 2 min of device calibration, 2 min of EEG recording and 3 min of eye movement recording. **(B)** The structure of the DBN model. The neuropsychological, EEG, and eye movement feature vectors were input. The features of raw input vectors were learned through RBM1 and RBM2 in order to classify MCI and healthy samples (Jiang et al., 2019b).

Based on the underlying characteristics of MCI including impaired visual attention and abnormal EEG rhythm, Jiang et al. designed a rapid and automatic MCI detection approach for primary care (Jiang et al., 2019a). This detection approach included a dynamic visual tracking task and an EEG recording task (Figures 8i). In the visual tracking trial, subjects were asked to gaze at a purple ball moving counterclockwise, following its moving direction and avoiding being disturbed by two other small balls. Eye movement features including the blink time, blink frequency, fixation time, and sustained attention span, were recorded during the trial. The EEG signals were detected and filtered by two dry sensors in the resting state. Forty features (12 eye movement-based features and 28 EEG-based features) were extracted by using linear and nonlinear analysis in the combined EEG and eye movement methods. The features associated with delta and alpha EEG dysrhythmia and impaired visual attention were screened by LR analysis. The final MCI screening model generated by the whole detection procedure had a high accuracy rate of 97.8%. This combined screening model can automatically complete the diagnostic tasks in just 5 min and suit for large-scale disease diagnosis, which is more efficient than the traditional lengthy test methods. Based on the above research, Jiang et al. (2019b) team further developed a Deep Belief Network (DBN) model (Figures 8ii) for more efficient early detection of MCI patients. DBN network consists of one input layer, several hidden layers, and one output layer with the functions of learning features and achieving classification. Feature extraction includes recording neuropsychological test scores and EEG and eye signal features using linear and nonlinear approaches in physiological tests. The collected eye dynamics and EEG-signal-based features were input into the DBN network, which were processed by two hidden layers to become more representative features. The output layer gave the ultimate result of whether the participant has MCI. In this study, the DBN model can select and classify features and simplify features in the hidden layer, which reached an 89.87% accuracy rate of detection results. Early screening for MCI has great significance in clinical application in primary care, which could reduce the number of people with AD or delay the development of AD.

Kang et al. (2020) designed a machine-learning approach to identify children with autism by eye-tracking data and EEG data. The subjects were 3 to 6-year-old children. Eye-tracking data were recorded when they gazed at faces of own race and other-race strangers. Eight areas of interest (AOI) including eye, left eye, eye, nose, mouth, whole face, body, and background were selected to quantify the fixation. Power spectrum analysis was used to detect the abnormal rhythm fluctuation of EEG in autistic children. The minimum-redundancy-maximum-relevance (MRMR) feature selection method was used for the feature selection of EEG, and the SVM classifier was combined to classify patients and normal controls. The results showed that the classification pattern combined the features of eye movement tracking and EEG had a higher classification rate than the single pattern, up to 85.44%. This study demonstrated that the multi-modal and multi-feature fusion classification approach provided a promising classification accuracy for future ASD diagnosis.

Many automatic detection methods based on biological signals have been proposed previously, however, their accuracy still needs to be improved. For practical clinical application, Zhu et al. (2019) further proposed a Content-Based Ensemble Model (CBEM) to enhance the accuracy of depression detection. The integrated model was composed of several classifiers. Two different experimental datasets were collected from the free-viewing eye-tracking task and the task-state EEG, respectively, and the stimulation consisted of different emotional faces. The data samples were divided into data subsets according to data types, and then the majority vote of the subsets was used to predict the labels of the subjects. The CBEM model applied to the two experiments separately achieved 82.5% accuracy (eye-tracking) and 92.73 percent accuracy (EEG). In general, CBEM achieved better classification results than traditional classification methods. Ding et al. designed another multi-modal machine learning method to diagnose depression. In a free-gaze task, participants viewed sets of images involving four emotional stimuli (positive, neutral, anxious, and threatening) (Ding et al., 2019). Tobii Eye-tracker 4C was applied to record visual gaze data. Frontal EEG signals were collected by MUSE EEG Headband, during which participants were required to watch eight short videos involving positive, neutral, and anxious emotions. Meanwhile, the Grove-GSR monitor, a biofeedback device, was used to record skin conductance. Galvanic skin response (GSR) is a fluctuation in skin electrical resistance caused by changes in sweat gland activity or the sympathetic nervous system and is often used to detect mood (Nourbakhsh et al., 2012). The features of three types of input data were extracted, and three machine learning algorithms, including RF, SVM, and LR, were used to establish the classification model. The f1 score is the harmonic average of the accuracy and recall of the binary classification analysis. According to the results, the highest f1 classification score was obtained by the LR algorithm, which achieved 79.63% accuracy, 76.67% precision, 85.19% recall, and 80.70% f1 score, respectively. This study integrated three physiological parameters and achieved a high classification score overall, suggesting that multi-modal fusion could improve the performance of the classification model.

The P300 waveform is a neuro-evoked potential component of EEG, which reflects the biological electrical activity generated in response to specific cognitive, sensory or motor events. P300 delay is sensitive towards cognitive impairment and can be used to detect schizophrenia (Zhang Y. et al., 2021c). Blackwood et al. (1994) used P300 auditory ERP and SPEM, two characteristic markers of schizophrenia, to test their association with schizophrenia and functional psychosis. A total of 20 families were recruited for the genetic linkage study, each with at least two diagnosed schizophrenics. Most households were found to have one or two anomalies in their measurements. P300 latency and eye-tracking measurements were normally distributed in schizophrenia patients and controls but bimodal in families. Abnormalities in SPEM and/or ERP occur in family members with schizophrenia, and about half of non-schizophrenic relatives present data abnormalities. This experiment demonstrated the potential role of indicators of psychophysiological abnormalities in genetic research.

In previous studies, traditional memory scales, named Wechsler Memory Scale (WMS), were used to evaluate the memory of patients with epilepsy (Wechsler, 1945). However, such scales failed to separate the effects of visual attention on the evaluation process, which also means that behavioral and associative processing are confused. To address this problem, Zhu et al. (2021) developed an automated computer-based platform for exploring the correlation between memory performance and visual attention in patients with TLE and separated the effects of visual attention on memory tasks by eye-tracker and EEG. The task consisted of a WSM assessment (Digital Span Backward and Visual Recognition tasks), cognitive oculomotor games, and all-day video EEG recordings. After analysis, the results showed that the TLE patients performed worse than normal subjects in the abilities of verbal memory and visual recognition. It was further confirmed that TLE patients spent longer searching for the target in the memory visual-stimulation games. And they also had more visit counts and first fixation time on the AOI during the recall process. Combined with the characteristics of bilateral temporal epileptic discharge (IEDs), the memory performance of TLE patients was negatively correlated with the temporal lobe peak during sleep. In general, patients with TLE have defects in short-term memory. A measuring platform combining a wearable eye-tracker and VEEG can be long-time monitoring of memory performance and visual attention in TLE.

Application examples for multi-signal sensors are listed in Table 5. Through combining multiple non-invasive neural signal recording methods, more mental diseases could be diagnosed. Besides, compared or combined more than two diagnostic methods by machine learning could improve the accuracy. For example, 97.8% accuracy was achieved in the diagnosis of MCI by combining eye-tracking data and EEG signals. Although the screening model integrated by multiple classifiers can complete the diagnosis of MCI in as little as 5 min. Essentially, the data collection process of multi-signal-based sensors is the same as that of a single sensor, still requiring long preparation and collection times and redundant steps. Therefore, the wearable multi-signal integrated sensors with shorter time consumption will have great potential to become a development trend in the future.

**TABLE 5:** Examples of multiple-signal sensors towards mental diseases diagnosis. DBN, deep belief network; CBEM, content-based ensemble model; AOI, areas of interest; ERP, event-related potential.

Bio-sensors	Mental diseases	Equipment/Technology	Diagnostic standards	Assessment standards	Results	Advantages	Ref
Multiple-signal sensors	Mild cognitive impairment	MindWave MW001 and Tobii TX-300	Visual attention processing and EEG rhythms	LR	97.8% accuracy	Allow head movement, automatic and quickly	Jiang et al. (2019a)
		MindWave MW001 and eye-tracker	Neuropsychological, EEG, and eye movement features	DBN	89.87% accuracy	Low-cost and automatic	Jiang et al. (2019b)
	Autism spectrum disorder	128-channel HydroCel Sensor Net System and Tobii TX300 eye-tracking system	Eight AOI areas towards faces of own race and other-race people	SVM	85.44% accuracy	Multi-feature and multi-modal fusion	Kang et al. (2020)
	Depression	EyeLink 1000 Eye Tracker and P300 stimulator	Various emotional faces	CBEM	92.73% accuracy	Higher accuracy of classification	Zhu et al. (2019)
		Tobii Eye-tracker 4C, MUSE EEG Headband, and grove-GSR monitor	Four emotional stimuli	SVM, RF, and LR	79.63% accuracy	Low-cost and portable	Ding et al. (2019)
	Schizophrenia	P300 stimulator and silver-silver chloride electrodes	The two-tone auditory discrimination and eye movement tasks	ERP and SPEM	Abnormalities occur in family members with schizophrenia	Proved that schizophrenia has a genetic component	Blackwood et al. (1994)
	Epilepsy	WMS, Tobii Glass II, and 580-G2CGSS VEEG monitoring system	WSM assessment, cognitive oculomotor games, and EEG recordings	memory performance and visual attention	Negatively correlated with the temporal lobe peak	Long-term monitoring	Zhu et al. (2021)

## 4 Conclusion and future perspective

In this review, biological pathways to diagnose mental disorders are classified into five categories: visual-based, EEG signal, EOG signal, VR-based and multiple-signal sensors. The vision-based sensors capture the signals of eye movements through eye-tracking devices. Both EEG and EOG signals sensors apply electrode arrays to capture signals. VR-based sensors collect user responses and behaviors by immersing them in a virtual 3D environment. The multiple-signal sensors are a combination of two to three above biosensors.

The vision-based sensors quantify the bias of attention by eye-tracking data. The rationale is to reveal the different attention-shifting mechanisms between patients and controls by comparing the time of gaze and disengagement in stimulus tasks (e.g., pictures, web pages, facial expressions, and communication) in two groups. Currently, eye-tracking technology can identify AD, depression, and ASD, which suggests that vision can be used as a promising biomarker to detect mental diseases. Patients with AD and depression showed a less attentional preference for novel and positive things in the experiments. Depressed patients spent more time disengaging from negative information, and the severity of the disorder was positively correlated with disengagement time. People with ASD tend to focus on details and ignore the whole. The accuracy rate of diagnosing ASD patients in face-to-face communication experiments reached 92.31%, indicating the potential of screening mental diseases in daily communication in the future. The vision-based method has the benefits of being non-invasive, easy to obtain data and wearable. However, a limitation of this method is that the inherent shaking and blinking of the eye make it difficult to extract accurate data. And the movement of the head is easy to cause data interruption. In addition to this, additional preparation time is necessary, e.g., the need for optimal lighting conditions. Patients wearing spectacles or contact lenses not being measured is also a problem and may affect the representativeness of the sample.

In addition to the vision-based method, patients with ASD and depression also can be diagnosed by EEG signals because of abnormal neuronal activity in their brains. And patients with epilepsy and schizophrenia can also be diagnosed by EEG. ASD patients exhibited stronger activation in the frontal, central, parietal, and occipital regions when viewing faces with different emotions than healthy controls, which is related to abnormalities in attention, emotion, and executive function. The acquisition of EEG data from the left and right hemispheres only requires the patient to be at rest for 10 min rather than visual stimulation. Due to the complex, non-stationary and nonlinear characteristics of EEG signals, several computer-aided diagnosis systems (CADS) were proposed to evaluate the data. The performance of CADS directly depends on how features are extracted and classified by machine learning techniques. The complexity of EEG signals could be reduced by fast Fourier transform and wavelet transform, etc. Moreover, machine learning methods, e.g., SVM and KNN can realize automatic classification of epilepsy and depression, with less computation and higher classification accuracy (99.3% ACC). And screening schizophrenia patients by RF classifier achieved 94% accuracy. In addition, two brain diseases (depression and epilepsy) could be diagnosed at the same time, which means the technology to screen for two or more mental disorders at the same time could be available in the future. However, the EEG signal-based method is not nearly perfect. For example, not all patients have abnormal EEG performance at all times. Patients may appear completely normal on short EEG tests, which can lead to errors in experimental results. Long-range EEG measurement is more conducive to detecting abnormal signals. But the disadvantage is that brain waves are easily disturbed by many factors. And the EEG signal of the patient is difficult to detect inactivity. Scalp EEG signal acquisition also faces more difficulties. For example, it is necessary to solve the obstruction caused by the hair so that it can stably collect signals.

EOG is a technique for measuring eye movement and eye position based on changes in retinal resting potential under light and dark adaptation. Wearing the EOG device requires only one silver chloride electrode on the skin of the inner and outer canthus. Compared with other electrical signal detection technologies described above, EOG is lightweight, wearable, and easy to operate. In addition to these advantages, EOG signals can be detected in patients with eye closure, as well as in young uncooperative patients or those with nystagmus. The method for diagnosing schizophrenic patients by EOG signals is based on the abnormal biomarkers of QET and SPEM. However, these methods were proposed about 20 years ago. As machine learning becomes a mainstream statistical tool, it is now more common to diagnose schizophrenia in combination with EEG signals. In contrast, research on the diagnosis of schizophrenia based on EOG signals has hardly progressed in recent years. The results confirmed that schizophrenic patients and their unaffected relatives showed significantly lower pursuit gain, and more patients with left-side extinction showed abnormal SPEM. An interesting finding is that the research progress in diagnosing sleep disorders is inverse to that of schizophrenia. The early diagnosis methods of sleep disorders were mainly based on EEG signals, but the progress is mainly based on EOG signals combined with deep learning in recent years. Sleep disorders are diagnosed by using EOG and machine learning methods to extract features and score different sleep stages. However, the process of detecting EOG requires a long period of dark adaptation and light adaptation, which is limited to lighting conditions. And EOG signals are less sensitive than electromyography (EMG).

By utilizing a range of visual, auditory, tactile, and olfactory stimuli, VR technology allows users to immerse themselves in a virtual environment, offering a unique method for diagnosing mental diseases. Innovative VR-based sensors addressed the challenges of diagnosing AD, ASD, depression, schizophrenia, and epilepsy, and focused on deficits in navigation and spatial memory, daily activities and attention, facial emotion recognition, and social skills. For identified these biomarkers, VR could provide an ideal virtual environment. Compared with other biosensors, VR had the advantages of being reproducible and programmable. In the virtual environment, researchers could easily control variables and repeat experiments, and also allowed the existence of interference or noise. In addition to visual stimuli, the VR-based tests also included auditory stimuli. Even the sense of smell could potentially be used in experiments, which was the advantage that several other sensors do not have. Due to the development of wireless HMDs and mobile electronic devices and wireless communication technology, VR-based 24-h medical monitoring applications would be a common trend. However, VR technology also had some disadvantages. The elderly people were prone to dizziness in the virtual environment, and it was difficult for them to master the use of joysticks, etc. A more ideal spatial interaction experience, such as implanting voice, gesture, and eye controls, or allowing users to adjust the degree of virtuality and reality are possible solutions.

The multiple-signal sensors combine features acquired by a variety of non-invasive neural signal recording methods (vision, EEG, and EOG), and automatically performs the screening of mental diseases through an ensemble model composed of multiple classifiers. This integrated approach can detect a wider range of diseases and avoid the drawbacks of a single approach. For example, biological signals that are not visually detectable can be detected by EEG. In addition, the verification of more than two detection methods can improve the accuracy of diagnosis to a certain extent. However, the greater variety of data collected means patients take longer in the diagnosis process. Therefore, more comfortable and integrated wearable devices will be a big trend in the future.

The results of this review can be utilized in other engineering sciences, e.g., multi-signal biosensors, wearable devices, machine learning, telemedicine, etc. The mental diseases are currently diagnosed based on different biomarkers. The biosensors acquired bio-signals from patients and healthy controls during the stimulation task. Multi-signal sensors based on integrated classifiers provide a more efficient and accurate screening mode. Although the screening model can automatically complete the diagnosis within 5 min, the collection of different data still requires multiple different sensors in different tasks, which makes the participants feel very tedious in the data collection process. In the future, more novel biosensors with higher accuracy, efficiency and integration will be developed. For example, it may be possible to add eye-tracking and EOG signal collection functions to VR/AR head-mounted display devices, so that multiple signals can be collected in one task and one device. In addition, due to the intermittent nature of the onset of mental diseases, a short diagnosis (10 minutes) is not representative of the daily onset of patients. With the promotion of micro biosensor devices, Wearable fabrics, flexible electronics, and other technologies, smaller and more comfortable wearable biosensors will achieve real-time monitoring throughout the day in daily life. In addition to the visual signals, eye signals, brain signals and VR reviewed in this paper, biosensors can also monitor many physiological parameters, e.g., lactic acid and glucose in sweat metabolites, electrocardiogram, and body temperature. A diagnostic approach that combines several biosensors may become mainstream in the future, leading to the diagnosis of a wider range of mental disorders. Combining deep learning and artificial intelligence methods, feature extraction and classification of physical and biochemical data of the human body are implemented to achieve automated and large-scale diagnosis and new technology for simultaneous screening of multiple mental diseases. Diagnosis methods based on EEG signals and multi-signals are more commonly combined with machine learning in recent years, including algorithms such as SVM, KNN, RF, and LR, etc., which provide a higher accuracy rate for the screening of mental diseases. Currently, all of the above diagnostic approaches are based in a laboratory setting. The development of radio communication technology has greatly supported the diagnostic functions associated with medical devices. Data can be sent directly from the medical testing device to a remote health center or hospital. Doctors will be able to conduct remote consultations and guidance through virtual reality technology, and patients wearing testing devices will be able to put on their own devices and perform the diagnosis process at home.
